# Permanent Rabi oscillations in coupled exciton-photon systems with *PT* -symmetry

**DOI:** 10.1038/srep19551

**Published:** 2016-01-21

**Authors:** Igor Yu. Chestnov, Sevak S. Demirchyan, Alexander P. Alodjants, Yuri G. Rubo, Alexey V. Kavokin

**Affiliations:** 1 Vladimir State University named after A. G. and N. G. Stoletovs, Department of Physics and Applied Mathematics, Vladimir, 600000, Russia; 2 ITMO University, St. Petersburg 197101, Russia; 3 Instituto de Energías Renovables, Universidad Nacional Autónoma de México, Temixco, Morelos, 62580, Mexico; 4 Russian Quantum Center, Moscow Region, Skolkovo, 143025, Russia; 5 University of Southampton, Physics and Astronomy School, Highfield, Southampton, SO171BJ, UK; 6 CNR-SPIN, Viale del Politecnico 1, I-00133 Rome, Italy

## Abstract

We propose a physical mechanism which enables permanent Rabi oscillations in driven-dissipative condensates of exciton-polaritons in semiconductor microcavities subjected to external magnetic fields. The method is based on stimulated scattering of excitons from the incoherent reservoir. We demonstrate that permanent non-decaying oscillations may appear due to the parity-time symmetry of the coupled exciton-photon system realized in a specific regime of pumping to the exciton state and depletion of the reservoir. At non-zero exciton-photon detuning, robust permanent Rabi oscillations occur with unequal amplitudes of exciton and photon components. Our predictions pave way to realization of integrated circuits based on exciton-polariton Rabi oscillators.

Nowadays, semiconductor microcavities with embedded quantum wells serve as a model system for fundamental studies of dynamic, coherent, nonlinear and quantum effects in nonequilibrium ensembles of bosonic quasiparticles—exciton-polaritons^1^,^2^. Various approaches to photonic information processing with the use of microcavity polaritons have been proposed^3^,^4^,^5^,^6^,^7^. In particular, polariton circuits, or neurons, have been theoretically descibed^8^,^9^ and then experimentally examined^10^ by Ballarini *et al*. at extremely low pump intensities. Although nonequilibrium exciton-polariton Bose-Einstein condensation has been already observed in many labs, the strong dissipation of exciton-polaritons limits wide application their condensates for logic operations. Even in highest quality microcavities the leakage of photons leads to dissipation effects occurring on the scale of tens of picoseconds. In particular, the fast decay and decoherence of exciton-polaritons is a reason for rapid attenuation of Rabi oscillations occurring between exciton and photon components of a polariton state, which represent a particular interest for quantum information applications. Recently, some of us have proposed a method to improve the coherence time of polariton Rabi oscillations by stimulated pumping of a Rabi oscillator from a permanent thermal reservoir of polaritons^7^. The increase of the coherence time of polariton Rabi oscillations in the presence of *cw* pumping has been experimentally observed^11^, and manifestations of this effect in spatial dynamics of exciton-polaritons have been revealed both experimentally^12^ and theoretically^13^. Recently, Voronova *et al*. has studied^14^ theoretically the non-linear regime of polariton Rabi oscillations and their interplay with exciton-photon Josephson oscillations.

Here we describe the experimental configuration allowing for observation of permanent Rabi oscillations in a driven-dissipative spinor exciton-polariton condensate in semiconductor microcavity. We study the regime where the microcavity system obeys parity-time 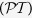 symmetry conditions. Originally, the 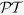-symmetry approach has been proposed^15^,^16^ by Bender *et al*. to demonstrate that some non-Hermitian Hamiltonians can be characterized by entirely real energy eigenvalues. In the recent decade, 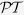-symmetry features have been demonstrated for various systems in photonics^17^,^18^,^19^,^20^,^22^,^23^, condensed matter physics^24^,^25^ and metamaterials^26^ and even electronic circuits^27^. Moreover, 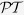-symmetry approach has been extended recently to a system of non-equilibrium low branch exciton-polariton condensates in micro-pillars weakly coupled to each other by tunelling^28^. Within the mean-field theory approach and neglecting local dispersion it was shown that the Hamiltonian of the couple may become 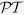-symmetric if injection of the polaritons at one site is equal to the decay at the other one. While this system has some formal similarity with the polariton Rabi-oscillator we consider here, there are also important differences. In the present work we study the interplay of exciton and polariton subsystems in one single coherent exciton-polariton system accounting for specific effects of detuning and blue-shift.

Physically, 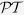-symmetry requirements can be easily understood by using the system of two linearly coupled oscillators^20^,^22^ (dimers, or waveguides in photonics ). The system obeys 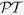-symmetry criteria if the rate of dissipation for one of the oscillators is exactly equal to the rate of gain in the other oscillator. This condition can be achieved in a system of two coupled polariton condensates with distributed dissipation rates rates^29^, that acquires a spectrum of eigen-states corresponding to real eigen-energies, that is reminiscent of a conservative system. In the presence of nonlinearity the physical picture in the system obeying 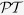-symmetry becomes richer and requires more complex analysis^21^. Particularly, for linearly coupled nonlinear oscillators the effect of nonlinearity-induced 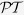-symmetry breaking occurs^22^. However, even in this case the time reversal symmetry leads to the balance between average gain and losses. Besides, it is important to note that even if the exact compensation between gain and losses in a two-mode system is not achieved, the concept of quasi-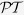-symmetry can be introduced^30^.

Here we demonstrate that the regime of permanent Rabi oscillations can be realized for the exciton-photon system in a microcavity, and the condition of 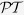-symmetry may be achieved if the gain in the excitonic component is compensated by losses from the photonic component.

## Basic equations

We consider a coupled exciton-photon system in the presence of the external magnetic field that produces the Zeeman splitting 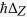 of exciton levels, and an incoherent excitonic reservoir, pumped by cw-field with the rate *P*. This non-resonant pumping can be realized both optically or by electronic current injection^31^,^32^. We assume that the wave function of driven-dissipative exciton-polariton condensate consists of photonic and excitonic components, 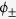 and 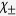. We will neglect the possible spatial degrees of freedom in what follows, assuming the condensate to be at zero momentum state. In this case, both components of the condensate and the reservoir can be described by Boltzmann kinetic equations:


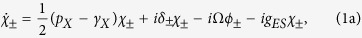



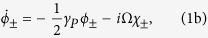



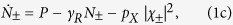


where dots denote time derivatives and 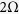 is a polariton Rabi splitting frequency. The subscript “+” (“−”) corresponds to the spin projection parallel (antiparallel) to the vector of magnetic field. In eq. (1) 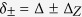 are effective photon-exciton detunings determined by the detuning 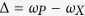 of the cavity mode and exciton frequencies in the absence of magnetic field and by the Zeeman splitting 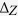; 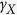, 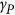 and 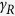 are the exciton, cavity and reservoir damping rates, respectively. The parameter 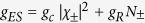 is responsible for exciton-exciton and exciton-reservoir interaction that induces a blue shift of the exciton energy. The most important features of Eq. (1) can be elucidated for the ideal gas of excitons assuming 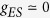 in Eq. (1). This approximation is justified in the vicinity of the pumping threshold, where both the exciton state population 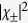 and the reservoir population *N* are not too large. Here we take into account the repulsive interaction between excitons of the same spin only.

In Eq. (1) the term containing 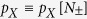 describes the pumping of the exciton state by stimulated scattering from the reservoir. We shall consider two possible scattering mechanisms neglecting scattering of excitons with opposite spins:


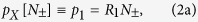



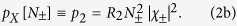


The Eq. (2a) implies the acoustic phonon assisted pumping with the rate^33^ 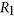, while Eq. (2b) describes the exciton-exciton scattering where excitons possessing momenta −**k** and **k** scatter into the condensate state with the momentum **k** = 0. Note that in both cases, the scattering feeds both upper and lower exciton-polariton branches. Our model accounts for the decay of both exciton and photon components of the condensate but neglects incoherent processes that lead to relaxation between upper and lower polariton branches.

Equations (1) can be solved for each of spin components separately. We first examine the subsystem with “+” spin component omitting the subscript for simplicity.

The dynamics of the exciton-photon system versus the pumping power demonstrates a threshold behavior at the specific value 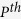 of the cw-pump intensity which is dependent on the particular mechanism of exciton pumping^33^ 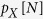. At 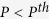 the exciton pumping rate 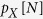 is comparable to or much lower than dumping rates 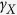 and 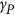 which, in turn, are much smaller than the Rabi splitting^34^ Ω. In this case one can assume the reservoir population *N* and the pumping rate 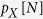 to be time independent on a time scale of the Rabi oscillation period ~Ω^−1^. In particular, for the pumping term given by Eq. (2a), eliminating Eq. (1c) we find eigenfrequencies of the exciton-photon system


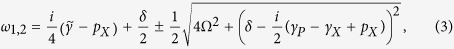


characterizing steady-state solutions 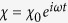 and 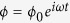. Physically, Eq. (3) determines frequencies of upper 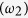 and lower 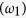 polariton branches^34^, measured with respect to the bare photonic frequency 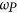. In Eq. (3) we denoted 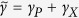. In this case, from Eq. (1c) one obtains 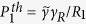.

The 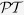-symmetry of the system (1) appears if the hermitian part of Hamiltonian is symmetric and the anti-hermitian part is anti-symmetric with respect to permutation of excitonic and photonic components. The first condition leads to


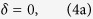


and the second condition requires that the losses from the photonic component to be fully compensated by the gain of the exciton one,


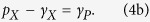


Clearly, if these conditions are satisfied the eigenfrequencies 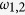 (3) become fully real. We note that in this case the amplitudes of oscillations of exciton and photon populations become equal 
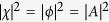
. Conditions (4a,b) can be experimentally realized by tuning of the pumping of the excitonic component with variation of the *cw* pumping strength *P*.

**Figure 1:**
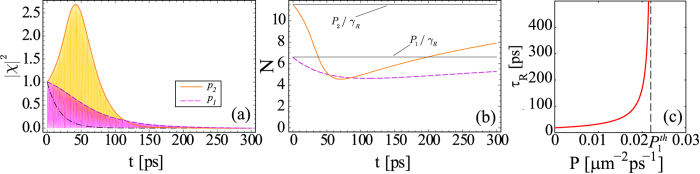
Time dependence of (a) the exciton population 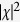 and (b) reservoir population *N* for 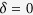 and under exciton state pumping 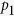 (magenta curve) and 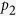 (yellow curve) for cw-pump *P* below threshold 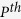 and (c)—Rabi oscillations lifetime 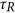 vs pumping rate *P* for the case of 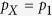 and 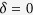. Dash-dotted black curve in (a) indicates decay dynamics in the absence of reservoir. Parameters are: 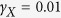 ps^−1^, 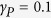 ps^−1^, 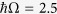 meV, 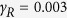 ps^−1^, 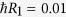 *μ*m^2^meV and 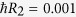 *μ*m^6^meV. Pumping rates are 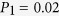 *μ*m^−2^ps^−1^ (magenta curve) and 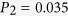 *μ*m^−2^ps^−1^ (yellow curve). Initial conditions are: 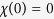, 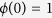, 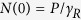.

Figure 1 reveals the decay dynamics of a coupled matter-light system described by Eq. (1) for different pumping mechanisms. The chosen parameters are realistic and correspond to experimentally accessible GaAs semiconductor microstructures^1^. In the paper for simplicity we represented excitonic 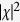 and photonic 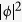 populations in the units of initial condensate density. As it is seen from Fig. 1a and follows from Eq. (3), the characteristic time 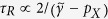 of Rabi oscillations decay can be increased by manipulating the pumping rate *P* (compare the dash-dotted black curve in Fig. 1a which is found in the absence of the reservoir and magenta and orange curves calculated accounting for the reservoir for two different values of the pump power). The dependence of lifetime 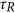 vs pumping rate *P* calculated by a numerical solution of Eq. (1) is shown in Fig. 1c. Starting from the value of 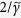, which corresponds to the polariton lifetime in the absence of reservoir 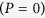, the lifetime 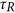 increases with the increase of the pump intensity. It diverges once *P* approaches the threshold value 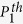. Starting from this point pumping fully compensates losses, i.e. the condition (4b) is fulfilled.

Thus, the condition (4b) represents a criterion for realization of permanent Rabi oscillations 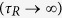. Such a regime cannot be achieved below threshold, at 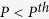. Actually, since the pumping rate 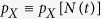 depends on the number of particles 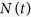 in the reservoir, it varies as a function of time, and the condition (4b) just cannot be preserved in the whole time interval. This indicates that the features of the combined exciton-photon system are strongly affected by the reservoir dynamics. At large time scales the reservoir population *N* approaches its stationary value 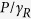
—see Fig. 1b, while exciton and photon mode population tends to zero.

In the case of exciton pumping predominantly governed by the exciton-exciton scattering process Eq. (2b), the dynamics of the system is more complicated. The orange curve in Fig. 1 demonstrates the behaviour of the envelope of exciton component oscillations [Fig. 1(a)] and reservoir population [Fig. 1(b)] below threshold 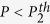 in this case. Although both components of the condensate are initially amplified (because of the reservoir depletion—see Fig. 1b), then the excitonic population 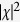 (and hence the pumping term 
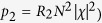
 attenuate very rapidly towards zero value. So, 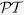-symmetry condition (4b) can not be fulfilled in this case.

## Results and Discussion

Now we analyze an important manifestation of the 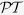-symmetry of the exciton-polariton system, namely, built up of the permanent Rabi oscillations regime at 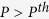.

**Figure 2:**
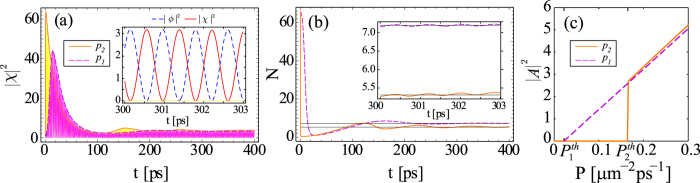
(**a**,**b**)—the same as in Fig. 1 but for cw-pump 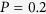 *μ*m^−2^ps^−1^ (for both curves), which is above threshold 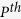 and (**c**)—the magnitude of Rabi oscillations 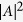 vs pumping rate *P*. Permanent Rabi oscillations for excitonic and photonic components, plotted on a zoomed time scale, are shown on the inset in (**a**). Relevant behavior of reservoir population is shown in the inset of (**b**).

We represent the solution of Eq. (1a,b) in the form


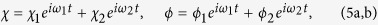


where 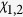 and 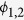 are constant amplitudes, which are proportional to the excitonic and photonic parts of the upper (index “2”) and lower (“1”) polariton states. In the regime of permanent oscillations, where the condition (4b) is fulfilled, an inequality 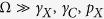 is satisfied. Thus, Rabi oscillations with the frequency proportional to Ω, occur essentially faster than the process of particle transfer from the reservoir to the exciton mode (whose rate is governed by 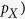. That is why, the reservoir population *N* remains approximately constant on a time-scale of the oscillation period. Actually, as it is seen from the inset in Fig. 2b, the reservoir population oscillates with a very low amplitude around its average value 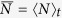 in the permanent oscillations regime. Thus, it is safe to assume the excitonic reservoir to be in the stationary state, 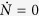.

At this point we would like to comment what we mean by “permanent” Rabi-oscillations. To start with, we work in the mean-field approximation that reduces the many-body quantum state of a Rabi-oscillator to a singly fully coherent state that can be described by a two-component wave-function. We do not consider any dephasing in the Rabi-oscillator, while it would necessarily spoil the mean-field picture sooner or later. Within the mean-field model we consider the amplification of a coherent seed set by the short laser pulse due to the stimulated scattering of polaritons from the incoherent reservoir. In a sense, this amplification process is similar to the coherence build-up in a polariton laser considered e.g. by^35^. In this spirit, we shall term the stationary regime obtained “permanent Rabi oscillations” if the coherent seed does not die out within the mean-field model.

### Permanent Rabi oscillations under phonon assisted pumping

Firstly, we examine the phonon assisted pumping of the exciton component of the condensate. Substituting Eqs (2a) and (5) into Eq. (1a,b) and separating real and imaginary parts we obtain


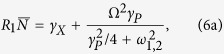



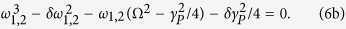


Eq. (6a) implies conditions of balance of pump and losses for upper and lower polaritons. Let us assume that the first requirement (4a) for the 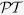-symmetry is fulfilled, i.e., we set 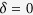. In this case, both upper and lower polariton states have equal exciton and photon components, i.e. 
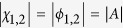
, and Eq. (6b) yields characteristic lower and upper polariton frequencies


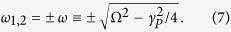


Equation (7) is familiar in 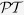-symmetry theory for coupled oscillators^18^,^19^,^20^. The value of 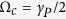 can be associated with the 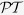-symmetry breaking threshold. In the case of a driven-dissipative exciton-polariton system, we operate significantly above the 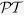-threshold point, assuming 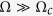, due to the strong exciton-photon coupling condition. Solution of Eq. (6a) with Eq. (7) leads to the condition


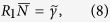


that simply implies the balance between pump and loss rates in the system and follows directly from the 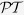-symmetry criterion (4b). The amplification of the magnitude of Rabi-oscillations due to the stimulated scattering of excitons from the reservoir saturates due to the depletion of the reservoir described by Eq. (1c).

Figure 2a demonstrates the establishment of the permanent oscillations for various mechanisms of the exciton pumping. The regime of permanent oscillations is reached after several hundreds of picoseconds for experimentally accessible parameters of the exciton-polariton system. Then exciton and photon populations oscillate out of phase (see the inset in Fig. 2a) with the equal constant amplitudes, determined by the relation


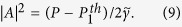


The amplitude 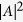 increases linearly with the increase of the pump power starting from the threshold point 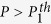 as it is shown by purple dashed line in Fig. 2c. This behavior is similar to one found in the driven-dissipative model of a polariton condensate in Ref. 33.

Undoubtedly, since the conditions (4) are satisfied now, this regime is also capable of having dynamical 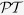-symmetry properties occurring at the same time scale. Notably, the establishment of permanent Rabi oscillations is accompanied by the quick reservoir depletion (see Fig. 2b) resulting in the sharp peak of exciton/photon populations for the first few picoseconds—see Fig. 2a.

For 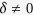 Eq. (6b) possesses three real roots, in general. Since the term 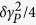 might be small enough for the moderate values of the detuning *δ* we can safely omit this term that allows to obtain [cf. (7)]


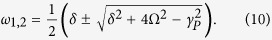


The value of 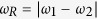 represents the frequency of Rabi oscillations. Since 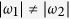, Eq. (6a) cannot be satisfied for both 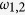 simultaneously. Physically, it means that upper and lower polariton branches are subject to different dissipation rates if 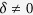. On the other hand, the pumping of both polaritonic states are equal and depends only on the reservoir population 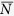. So it can not compensate losses of both polariton states simultaneously. That is why permanent oscillations supported by phonon assisted scattering processes cannot be realized in the nonresonant pumping case. This statement is in agreement with the first 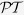-symmetry criterion (4a).

### Permanent Rabi oscillations under pumping by exciton-exciton scattering

Now let us examine the permanent oscillations regime for the exciton state pumping by exciton-exciton scattering. In this case, using Eqs. (5) and omitting higher-order harmonics we obtain


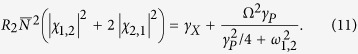


Notably Eq. (6b) is valid in this case as well and the frequency 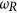 of oscillations is still determined by Eq. (10).

The limit of 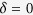 is similar to the one discussed in the previous subsection. The dynamics of the population of the excitonic component 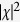 in this case is shown by the yellow curve in Fig. 2a. In this case we obtain a simple balance condition 
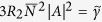
 of realization of permanent Rabi oscillations (as follows from Eq. (11)), where the amplitude of Rabi oscillations 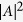 depends linearly on the pumping power above threshold 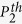 as the orange curve in Fig. 2c shows. The population 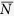 of the excitonic reservoir in the permanent oscillations regime reaches its stationary value (black horizontal lines in Fig. 2b) that can be found from the equation


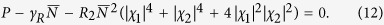


The main advantage of the discussed mechanism of exciton state pumping is that it can support permanent oscillations in the case of non-zero detuning, 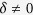. This is because Eqs. (11) admit solutions simultaneously for both 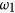 and 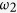 in the case of unequal amplitudes, 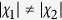. Actually, in the nonresonant case both upper and lower polariton modes again undergo nonequal losses. But due to the nature of the exciton mode pumping mechanism (2b) such losses can be separately compensated by the scattering from the reservoir. Unlike the case of phonon assisted processes, the rate of scattering into polariton states depends now not only on the reservoir density 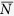 but also on the exciton fractions in the relevant polaritonic branches 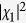 and 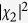. Suppose that the polariton state which is less exciton-like is subject to a stronger losses [see Eqs (10) and (11)]. Nevertheless, the efficient scattering stimulated by the excitonic part of another polariton state would allow for reestablishing the balance between pump and losses. That is why the resonance condition 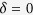 is not necessary in the case of exciton pumping predominantly governed by the exciton-exciton scattering process.

In spite of the fact that the criterion of the 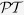-symmetry is not fulfilled in the nonlinear pumping case, the eigenfrequencies of the system 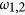 still remain purely real even in this regime. It means that in the presence of exciton-exciton scattering from the reservoir our system still exhibits properties of a pseudo-Hermitian system, that is characteristic of the regime of permanent Rabi oscillations.

It is important to underline that the compensation mechanism mentioned above would be efficient only if the value of detuning is smaller than some critical value 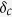. In fact, if 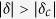 the population of an upper or a lower polariton branch (depending on the sign of *δ*) in the steady state regime vanishes, that indicates the collapse of Rabi oscillations. The value of 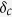 can be obtained using Eqs (10) and (11), and it reads approximately


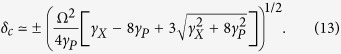


It is important that 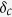 is governed by the parameters of a Rabi oscillator as a whole and does not depend on the parameters of the reservoir, including the external pump power *P*. For GaAs-based semiconductor microcavities we obtain 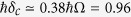 meV. Thus, permanent oscillations may be established in the vicinity of exciton-photon resonance for experimentally accessible parameters.

### Accounting for nonlinear energy-shifted processes

Let us examine the influence of the exciton-exciton scattering processes on permanent Rabi oscillations. It is possible to demonstrate that for weakly interacting excitons the term containing 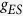 in Eq. (1) does not play a dominant role in Eqs (6a,b) and (11). In this case one can replace the effective detuning *δ* with its rescaled value


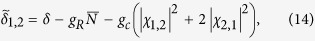


which now implies different energy shifts for polaritons of the upper and the lower branches.

**Figure 3:**
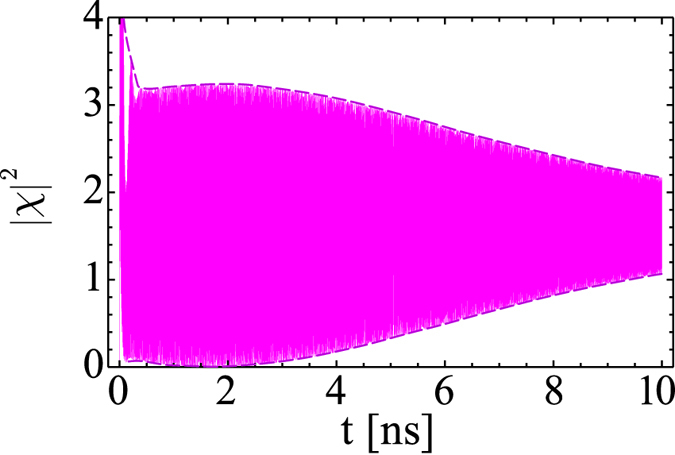
Relaxation of Rabi oscillations of the excitonic component due to the interactions between excitons for *p*_*X*_[*N*] = *p*_1_. System parameters are the same as in Fig. 2, excepting 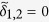 (see comments in the text). Besides, 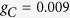 *μ*m^2^ps^−1^, 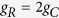. The vertical axis range was chosen to focus on permanent oscillations dynamics and does not show a sharp and high jump of 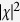 at first few picoseconds.

The first 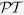-symmetry criterion (4a), which now writes as 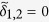, can be easily reached by an appropriate choice of the detuning 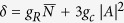, where 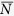 and 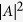 are given by Eqs (8) and (9) in the limit of phonon assisted exciton pumping (2a). Nevertheless, numerical simulations show that Rabi oscillations are characterized by lifetimes comparable or longer than the lifetime of excitons in semiconductor structures under study—see Fig. 3. The decay of Rabi-oscillations takes place because the exciton energy blue-shift is actually not constant in time: everywhere above the threshold it oscillates with the Rabi frequency because of the exciton density 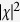 oscillations. The depletion rate of the reservoir and, consequently, the reservoir density *N* oscillate as well (Fig. 2b). Such a behavior dramatically affects the magnitude of Rabi oscillations supported with phonon-assisted mechanism, leading to the preferential pumping of one of two polariton branches and to the decay of oscillations, eventually. However, as it was previously shown, the exciton-exciton scattering mechanism (2b) still allows the establishment of the regime of permanent Rabi-oscillations with a magnitude dependent on the value of the detuning and exciton-exciton interaction constant. Fulfilment of the resonant condition for newly defined detunings (14) does not play a crucial role in this case. Numerical analysis demonstrates that exciton-exciton scattering affects the range of detunings where the permanent oscillations exist, see Eq. (13) and discussion in the previous subsection.

### Polarization properties

Here we consider a complete set of Eq. (1), that characterizes spin dependent exciton-polariton system. Polarization properties of the excitonic system are conveniently described by the components 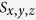 of the Stokes vector **S** defined by


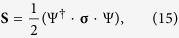


where 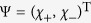 and 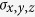 are the Pauli matrices. Since in general 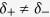 the behavior of Stokes vector components is completely determined by detuning Δ for a given non-zero Zeeman shift 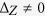.

**Figure 4:**
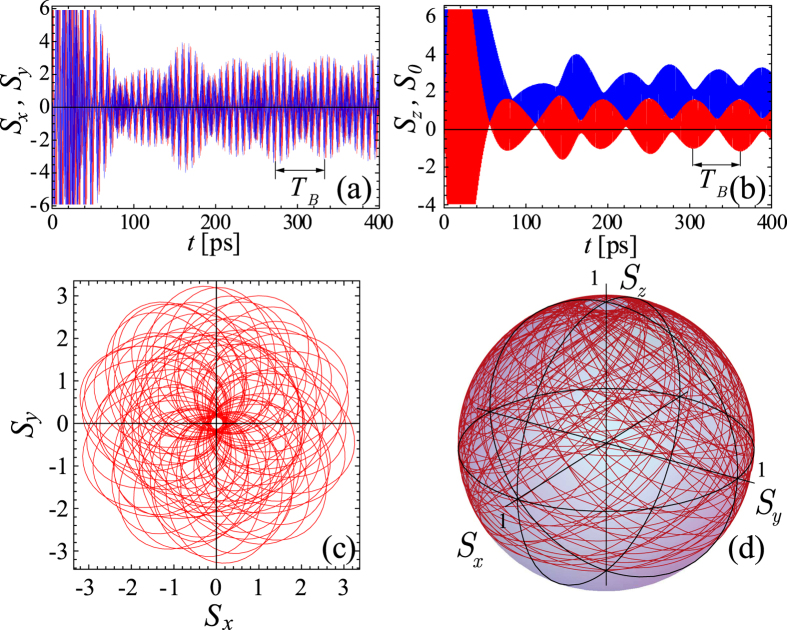
Time evolution of Stokes parameters for Δ_*Z*_ = 0.2Ω, Δ = 0.15Ω for the case of the exciton state pumping by exciton-exciton scattering (*p*_*X*_[*N*] = *p*_2_). The panel (**a**) shows the behavior of 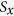 (red) and 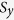 (blue) components, while 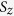 (red) and 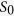 (blue) component is shown in the panel (**b**). The evolution of polarization on 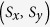-plane and on Poincare sphere on the time scale of 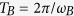 characterizing permanent Rabi oscillations beating period is shown in the panels (**c**,**d**), respectively. The parameters are the same as in Fig. 1, excepting *P* = 0.2 *μ*m^−2^ ps^−1^.

**Figure 5:**
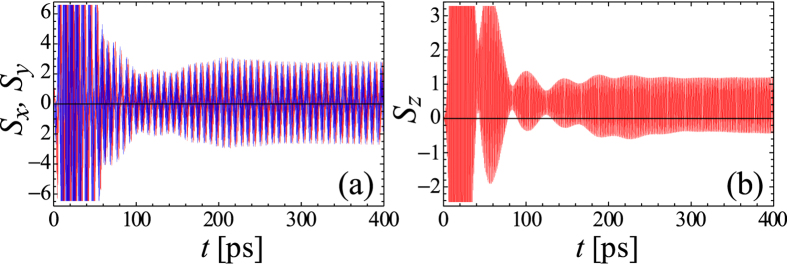
Time evolution of Stokes parameters *S*_*x,y,z*_ for Δ_*Z*_ = Δ = 0.2Ω for the case of phonon-assisted pumping (*p*_*X*_[*N*] = *p*_1_). The parameters are the same as in Fig. 4.

The general case corresponding to both Δ and 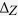 are nonzero is illustrated by Fig. 4. Using ansatz (5) and definition for Stokes vector (15) it is easy to show that components 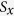 and 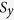 oscillate with the frequency 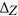 (frequent beats in Figs 4a and 5a). Besides there are fast oscillations with the frequency 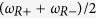 close to the frequency of Rabi oscillations. If the conditions of establishing of permanent oscillations are fulfilled for both spin components simultaneously 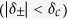, the frequencies of Rabi oscillations 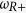 and 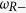 are different, because of the relation 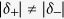. In this case, the amplitudes of oscillations of Stokes vector components undergo additional long-period beats with the period 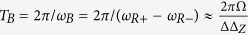 (shown on Fig. 4a,b). These beats occurs for all 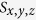 components and are sustained in time.

In Fig. 4d we represented the evolution of the Stokes vector **S** normalised to the total number of excitons 
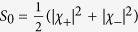
 on the Poincare sphere with a unit radius. The value 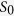, describing the excitonic subsystem, oscillates in time as shown in Fig. 4b. Note, that due to the oscillation of all components of Stokes vector, it spans almost the whole Poincare sphere, that is similar to the result obtained in the recent paper^36^ by two-pulse excitation in the absence of magnetic fields.

Let us consider the limit where permanent oscillations disappear for one of the spin components, i.e. for 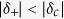 and 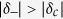, or vice versa, that can be achieved by the appropriate choice of 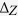 and 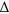. In fact, in this case the beats in Stokes parameters with the period 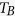 are suppressed in time and the amplitude of oscillations tends to the constant value. Figure 5 demonstrates these features for Stokes parameters in the specific case of 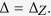 In this limit, the detunings 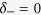 and 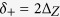. Physically, it means that permanent oscillations can be established for an exciton pumping mechanism described by (2a), but only for “−” spin component while population of another component will approach a constant value. At long time intervals only fast oscillations of Stokes vector components survive.

## Conclusions

We have described the regime of permanent Rabi oscillations in a driven-dissipative exciton-photon system feeded from the incoherent exciton reservoir. Two types of reservoir-Rabi-oscillator coupling mechanisms have been examined. We have shown that the permanent Rabi oscillation regime may be realized above the threshold pumping where the dynamical 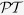-symmetry of the coupled exciton-photon system is achieved. Notably, weak interactions between excitons disturb the permanent character of Rabi oscillations within time scale much exceeding the period of Rabi oscillations in the case of phonon assisted exciton state pumping processes. However, it is important that such oscillations may be obtained by appropriate engineering of coupling between condensate and excitonic reservoir. In particular, the robust permanent Rabi oscillations regime occurs in a non-resonant case only if the exciton component of the condensate is predominantly pumped by exciton-exciton scattering. We studied excitonic polarization properties in the presence of external magnetic fields. The conditions for permanent Rabi oscillations for polarized excitonic or photonic system are established and discussed. Realization of permanent Rabi oscillations in this case play a crucial role for creation and manipulating of long-lived spin polarisation in the exciton-photon system which pave the way to the design of new optical memory devices.

## Additional Information

**How to cite this article**: Chestnov, I. Y. *et al*. Permanent Rabi oscillations in coupled exciton-photon systems with 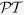-symmetry. *Sci. Rep.* **6**, 19551; doi: 10.1038/srep19551 (2016).
